# Low Molecular Weight Heparin Relieves Experimental Colitis in Mice by Downregulating IL-1β and Inhibiting Syndecan-1 Shedding in the Intestinal Mucosa

**DOI:** 10.1371/journal.pone.0066397

**Published:** 2013-07-18

**Authors:** Xian-fei Wang, Ai-ming Li, Jing Li, Shi-yong Lin, Chu-di Chen, You-lian Zhou, Xia Wang, Cun-long Chen, Si-de Liu, Ye Chen

**Affiliations:** 1 Guangdong Provincial Key Laboratory of Gastroenterology, Department of Gastroenterology, Nanfang Hospital, Southern Medical University, Guangzhou, China; 2 Department of Gastroenterology, Affiliated Hospital of North Sichuan Medical College, Nanchong, China; Baylor College of Medicine, United States of America

## Abstract

Low molecular weight heparin (LMWH) exhibits anti-inflammatory properties, but its effect on inflammation in colitis remains unclear. This study aimed to evaluate the therapeutic effects of LMWH on dextran sulfate sodium (DSS)-induced colitis in mice, in which acute colitis progresses to chronic colitis, and to explore the potential mechanism involved in this process. C57BL/6 mice were randomly divided into control, DSS, and DSS plus LMWH groups (n = 18). Disease activity was scored by a disease activity index (DAI). Histological changes were evaluated by hematoxylin and eosin (HE) staining. The mRNA levels of syndecan-1, interleukin (IL)-1β, and IL-10 were determined by quantitative reverse transcription polymerase chain reaction. Protein expression of syndecan-1 was detected by immunohistochemistry. The serum syndecan-1 level was examined by a dot immunobinding assay. LMWH ameliorated the disease activity of colitis induced by DSS administration in mice. Colon destruction with the appearance of crypt damage, goblet cell loss, and a larger ulcer was found on day 12 after DSS administration, which was greatly relieved by the treatment of LMWH. LMWH upregulated syndecan-1 expression in the intestinal mucosa and reduced the serum syndecan-1 level on days 12 and 20 after DSS administration (*P*<0.05 vs. DSS group). In addition, LMWH significantly decreased the expression of both IL-1β and IL-10 mRNA on days 12 and 20 (*P*<0.05 vs. DSS group). LMWH has therapeutic effects on colitis by downregulating inflammatory cytokines and inhibiting syndecan-1 shedding in the intestinal mucosa.

## Introduction

Low molecular weight heparins (LMWHs) are mixtures of heparin molecules in the range of 3000 to 10,000 Daltons [1]. LMWH is widely used as an anticoagulant drug based on its antithrombin III-activating properties as unfractionated heparin. Recently, LMWH has been found to possess anti-inflammatory properties [2]. Moreover, it has been demonstrated that LMWH ameliorates the inflammatory response in experimental colitis in rats [3], [4], [5] and syndecan-1-deficient mice [6]. Accumulating evidence has indicated that LMWH is beneficial for treating patients with active ulcerative colitis [3], [4], [5], while the underlying mechanism remains poorly understood.

Syndecan-1 is a member of the syndecan family, which consists of the cell-surface heparan sulfate proteoglycans (HSPG) that regulate cell behavior by binding extracellular matrix molecules such as growth factors, cytokines, and even heparin-binding proteins on the bacterial surface [7]. Syndecan-1 plays an important role in promoting wound repair, maintaining cell morphogenesis, and mediating inflammatory responses [8]. In addition, syndecan-1 participates in the composition of tight junctions and maintains mucosal barrier function [9]. Interestingly, recent studies have shown that syndecan-1 is shed and released into the blood and that syndecan-1 shedding is an important mechanism by which neutrophilic inflammation is relieved by aiding the clearance of proinflammatory chemokines in a heparan sulfate-dependent manner [10], [11], [12]. Activated syndecan-1 shedding is observed in many pathological processes such as inflammatory response in the lung [13].

In the present study, we investigated the effects of LMWH on dextran sulfate sodium (DSS)-induced colitis in mice, in which acute colitis progresses to chronic colitis. LMWH is an analogue of syndecan-1 and can competitively inhibit or imitate the function of syndecan-1. Thus, we hypothesized that syndecan-1 may be involved in the inflammation of colitis.

## Materials and Methods

### Reagents

DSS (molecular weight, 36–44 kDa) was obtained from MP Biomedicals, USA. LMWH (enoxaparin) was purchased from Aventis, France. TRIzol reagent was purchased from Invitrogen (Foster City, CA, USA). The qRT-PCR kit was obtained from Fermentas (Hanover, MD, USA). The monoclonal rat anti-mouse syndecan-1 antibody (281–2) was provided by Dr. Pyong Woo Park (Children's Hospital of Harvard University, Boston, MA, USA). Horseradish peroxidase (HRP)-conjugated goat anti-rat antibody was from Zhongshan Jinqiao Biotechnology (Beijing, China). The polyvinylidene difluoride (PVDF) membranes were purchased from Millipore (Bedford, MA, USA).

### Animals and grouping

A total of 54 male C57BL/6 mice (6–8 weeks old, 16–18 g in weight) were obtained from the Animal Center of Southern Medical University (Guangzhou, China). All mice were housed in specific pathogen-free (SPF) conditions and had free access to food and water prior to the experiment. The mice were randomly divided into three groups: control, DSS, and DSS plus LMWH (n = 18). Colitis was induced as previously reported [14], [15]. Mice in the DSS and DSS plus LMWH groups were given 3% DSS solution dissolved in distilled water for 5 days followed by 14 days of feeding with distilled water. In the DSS plus LMWH group, mice received daily subcutaneous injection of LMWH (5 μg/mouse) for 19 days. In the control group, mice were given fresh distilled water and an injection of the same volume of normal saline every day. The protocols for animal experiments were approved by the Ethics Committee of Southern Medical University (Permission No.: 2009–015).

### Evaluation of colitis

The disease activity index (DAI) was determined by scoring the extent of body weight loss, stool hemoccult positivity or gross bleeding, and stool consistency in accordance with the method described by Murthy *et al*. [16] (Table 1). The DAI was assessed by an investigator who was blind to the experimental groups.

**Table 1:** Disease activity index of colitis.

Score	Weight loss (%)	Stool* consistency	Occult/gross bleeding
0	(–)	Normal	Normal
1	1–5		
2	5–10	Loose	Guaiac (+)
3	11–15		
4	>15	Diarrhea	Gross bleeding

The disease activity index = (combined score of weight loss, stool consistency, and bleeding)/3.

* Normal stools: well formed pellets; loose: pasty stools that do not stick to the anus; diarrhea: liquid stools that stick to the anus.

### Histological and immunohistochemical analyses

Six animals from each group were sacrificed by subcutaneous injection of a lethal dose of 10% chloral hydrate for further examination on days 5, 12, and 20. Half of the samples were subjected to histological study and immunohistochemical analysis,the rest were used to measure mRNA expression of mucosal Syndecan-1, IL-1, and IL-10. The colonic tissues were excised and rinsed with ice-cold phosphate-buffered saline. Some colonic tissues were fixed in 4% paraformaldehyde (PFA), dehydrated with a graded ethanol series, cleared in dimethylbenzene, and embedded in paraffin. Hematoxylin and eosin (HE) staining was performed according to standard procedures. Some of the sections were subjected to immunohistochemical analysis using a monoclonal anti-syndecan-1 antibody (clone 281–2) and an immunohistochemistry kit (Kit-5001) according to manufacturer's instructions (Maixin Bio Fuzhou, China). The images were captured by a Nikon Eclipse E600 microscope (Tokyo, Japan). The rest tissues were snap frozen in liquid nitrogen and stored at −80°C for RNA isolation.

### Histological score

The histological score was a combined score of acute inflammatory cell infiltration (0–4), chronic inflammatory cell infiltration (0–3), and crypt damage (0–4), as described previously [15]. Specifically, the crypt damage was scored in the following manner: 0 for an intact crypt, 1 for loss of the basal one-third of the crypt, 2 for loss of the basal two-thirds of the crypt, 3 for entire loss of the crypt, and 4 for loss of the crypt and surface epithelium. At least 15 areas on each slide were examined, and the average score was calculated.

### Immunohistochemical score

Immunostaining for syndecan-1 on the membranes of intestinal epithelial cells and glandular epithelial cells was semi-quantified by incorporating both the intensity and the distribution of staining, yielding a subjective score as previously described [17]. Briefly, staining intensity was graded according to the following criteria: 0 (no staining), 1 (weak staining = light yellow), 2 (moderate staining = yellowish brown), and 3 (strong staining = brown). The average percentage of positive cells was converted into a four-tier numerical score (0.1, <25%; 0.4, 25–50%; 0.6, 51–75%; 0.9, >76%). The average score was calculated from at least eight sections for each sample. The total score was then generated based on the average staining intensity and the average percentage of positive cells.

### Quantitative real-time polymerase chain reaction analysis

Total RNA was extracted from frozen intestinal tissues with TRIzol reagent (Invitrogen, USA). The OD260/280 values were measured with a spectrophotometer (Bio-Rad, Hercules, CA) to determine the RNA concentrations. A High Capacity cDNA Reverse Transcription Kit (Applied Biosystems, Foster City, CA, USA) was used to synthesize cDNA from 1 ug RNA following the manufacturer's recommendations. qRT-PCR was performed to detect the expression levels of syndecan-1, IL-1β, IL-10 and GAPDH. Amplification mixtures for qRT-PCR contained 20 ng template cDNA, 10 ul Taqman Master Mix (Applied Biosystems) and probes in a final volume of 20 ul. The sequences of the primers were as follows: syndecan-1 upstream (5′-CAGCAGCAACACCGAGAC-3′) and downstream (5′-CTCCTACCTTGACGGTTAG-3′); IL-1β upstream (5′-TCATTGTGGCTGTGGAGAAG-3′) and downstream (5′ -AGGCCACAGGTATTTTGTCG-3′); IL-10 upstream (5′ -TGCTATGCTGCCTGCTCTTA-3′) and downstream (5′ -TCATTTCCGATAAGGCTTGG-3′); GAPDH upstream (5′ -TGATGACATCAAGAAGGTGGTGAAG-3′) and downstream (5′ -TCCTTGGAGGCCATGTGGGCCAT-3′). PCR was performed for 40 cycles with denaturation (95°C for 30 s) and annealing (62°C for 34 s). Specificity of amplification was checked by melt curve analysis. mRNA expression for the different genes was normalized against GAPDH and fold change over control was determined according to the ddCt method described previously [18].

### Dot immunobinding assay

The shed syndecan-1 in the serum was detected by a dot immunobinding assay. Before animals were sacrificed, blood samples were collected from the apex of the heart using a 1 mL syringe. The serum was separated, and 2 μL of the serum sample was loaded onto the dot-blot apparatus (Minifold, USA). In the control group, ABS-Tween was used instead of the serum sample. The PVDF membranes were blocked in 5% non-fat milk in Tris-buffered saline (TBS) for 1 h at room temperature. After washing with TTM solution twice, the membranes were probed with syndecan-1 antibody (0.2 μg/mL dissolved in TTM solution) at 4°C overnight. The membranes were then washed twice in TTM for 30 min each time and incubated with HRP-conjugated goat antibody (1 mg/ml in TTM) for 2 h at room temperature. After washing twice, the dots were visualized by incubation of the membranes in ECL solution and then exposed. Finally, the IOD value was quantified.

### Statistical analysis

Statistical analysis was performed using SPSS13.0 software (SPSS Inc., Chicago, IL, USA). Data were expressed as means±standard deviation (SD). One-way analysis of variance (ANOVA) analysis was used to assess within group differences. One-way ANOVA or the independent-sample t-test was used to determine the differences among different groups. Significance was determined at *p<*0.05.

## Results

### LMWH ameliorates colitis in mice

**Figure 1:**
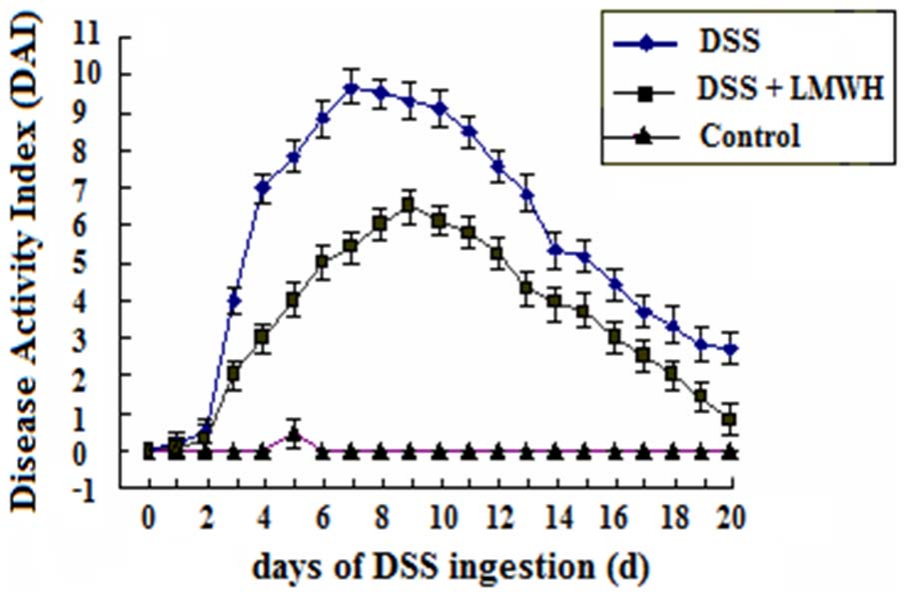
Disease activity index (DAI) of colitis in mice from different experimental groups. The DAI value was calculated as described in Materials and Methods (n = 18/group). *P*<0.05, DSS plus LMWH or DSS groups vs. the control group from day 3 to day 20 after DSS administration; *P*<0.05, the DSS plus LMWH group vs. the DSS group from day 3 to day 20 after DSS administration.

Mice in the control group showed normal body weight and stools (Table 1). However, in the DSS group, diarrhea and guaiac positivity appeared on day 3 and gross bleeding occurred on day 4 after DSS administration. Two weeks later, animals presented chronic colitis with signs of loose stools and guaiac positivity. Compared with control mice, the DAI value was significantly greater from days 3 to 20 in mice treated with DSS (*P*<0.05, Fig. 1). Compared to the DSS group, the colitis symptoms were dramatically relieved on day 6 in mice of the DSS plus LMWH group. Additionally, LMWH treatment significantly decreased the DAI value compared with the DSS group beginning on day 6 (*P* = 0.023, Fig. 1).

### LMWH improves the histological disturbances of colitis induced by DSS

**Figure 2:**
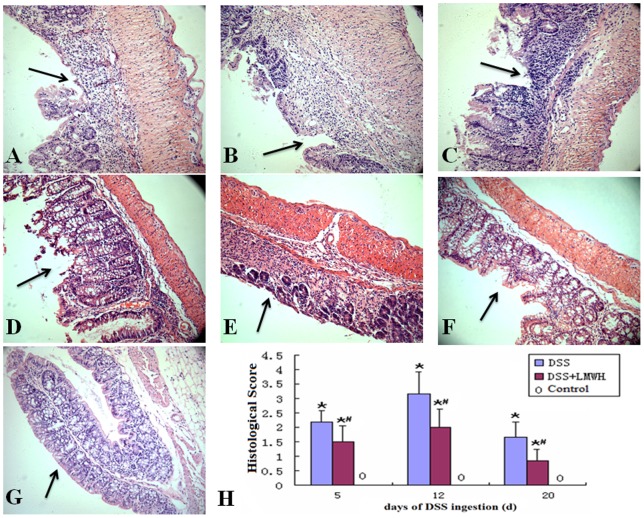
Histological analyses of the colonic tissues. The distal ends of the colon were fixed, embedded, sectioned, and stained with HE. Pathological features were indicated by arrows. A-G: Representative images show the histological structures of the colonic tissues in the DSS group (A, day 5; B, day 12; C, day 20) and the DSS plus LMWH group (D, day 5; E, day 12; F, day 20). Normal colonic tissues were used as the control (G). Original magnification: ×200. H: Quantization of histological scores (n = 18/group). *P<0.05 vs. the control; #*P*<0.05 vs. the DSS group.

Next, we examined the histology of the intestinal mucosa in different experimental groups. The distal ends of the colon obtained from each group were stained with HE. After 5 days of DSS administration, impaired intestinal mucosa, crypt, and gland damage, a multifocal shallow ulcer, as well as neutrophil infiltration of the epithelium and lamina propria appeared (Fig. 2A). Similar but slightly relieved histological disturbances were observed in mice treated with DSS plus LMWH on day 5 (Fig. 2D). Aggregative colon destruction with the appearance of crypt damage, goblet cell loss, and a larger ulcer were found on day 12 after DSS administration (Fig. 2B), which was greatly relieved by the treatment of LMWH (Fig. 2E). Notably, the symptoms of colitis were relieved on day 20 after DSS administration, showing limited multifocal aphthae, crypt, and goblet cell regeneration, gland repair, as well as lymphocyte and monocyte infiltration of the mucosa and submucosa (Fig. 2C). In the DSS plus LMWH group, LMWH remarkably decreased the histological disturbances on day 20 compared with the DSS group (*P* = 0.038, Fig. 2F). Intact intestinal mucosa and intestinal epithelium as well as a regular gland array were detected in control mice (Fig. 2G). Neither inflammatory cell infiltration nor an ulcer was found in these mice. Quantification of the histological score in different groups indicated that LMWH significantly reduced the histological disturbances on days 12 and 20 after DSS administration (*P*<0.05 compared to the DSS group).

### LMWH inhibits syndecan-1 shedding in the intestinal mucosa of DSS-induced colitis

**Figure 3:**
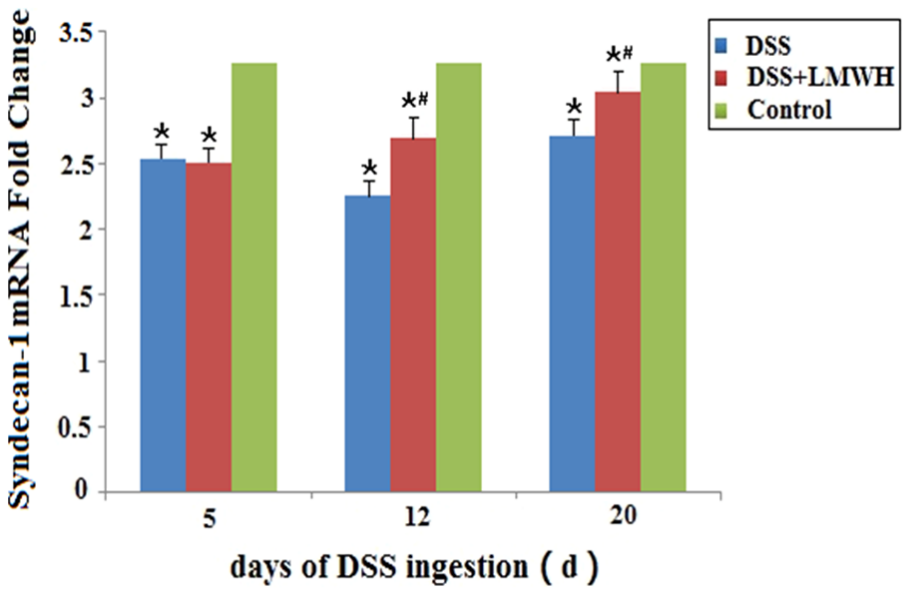
mRNA expression of syndecan-1 in the intestinal mucosa. The mRNA level of syndecan-1 in the intestinal mucosa was evaluated by qRT-PCR on the indicated day respectively. (n = 18/group). *P<0.05 vs. the control; #*P*<0.05 vs. the DSS group.

To evaluate the involvement of syndecan-1 in LMWH-mediated alleviation of colitis, we examined the expression of syndecan-1 in the intestinal mucosa at both the mRNA and protein levels by qRT-PCR and immunohistochemistry, respectively. In the DSS group, the mRNA expression of syndecan-1 in the intestinal mucosa was significantly less on days 5, 12, and 20 compared with that in the control group (*P*<0.05, Fig. 3). However, the mRNA level of syndecan-1 was greater on days 12 and 20 in the DSS plus LMWH group, compared to that in the DSS group (*P*<0.05, Fig. 3).

**Figure 4:**
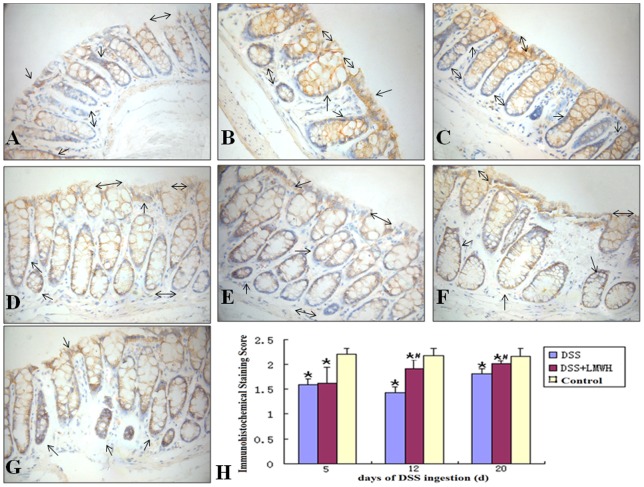
Protein expression of syndecan-1 in the intestinal mucosa. A–G: The tissue sections were subjected to immunohistochemical analysis with the syndecan-1 antibody. Syndecan-1 was expressed on the membranes of epithelial cells and gland cell membranes (single arrow head) in control mice (G). A marked staining reduction was detected in mice of the DSS group (A, day 5; B, day 12; C, day 20; double arrow heads). Partial epithelial membranes and gland cell membranes are moderately stained in colon mucosa of mice in the DSS plus LMWH group (D, day 5; E, day 12; F, day 20; double arrow heads). Original magnification: ×400. H: Quantization of sydecan-1 immunostaining (n = 18/group). *P<0.05 vs. the control; #*P*<0.05 vs. the DSS group.

Next, we performed immunohistochemical staining of syndecan-1 in the intestinal mucosa. In the control group, almost all membranes of epithelial cells and glandular epithelial cells were stained as dark yellow, while in the DSS group only some of the cellular membrane was stained yellow. In addition, the immunohistological score was significantly less in the DSS group compared to that in the control group (*P*<0.05, Fig. 4). However, in the DSS plus LMWH group, on days 12 and 20 after DSS treatment, most cellular membranes were stained dark yellow and the immunohistological score was significantly greater than that in the DSS group but still less than that in the control group (*P*<0.05 vs. the DSS group; *P*<0.05 vs. the control, Fig. 4).

**Figure 5:**
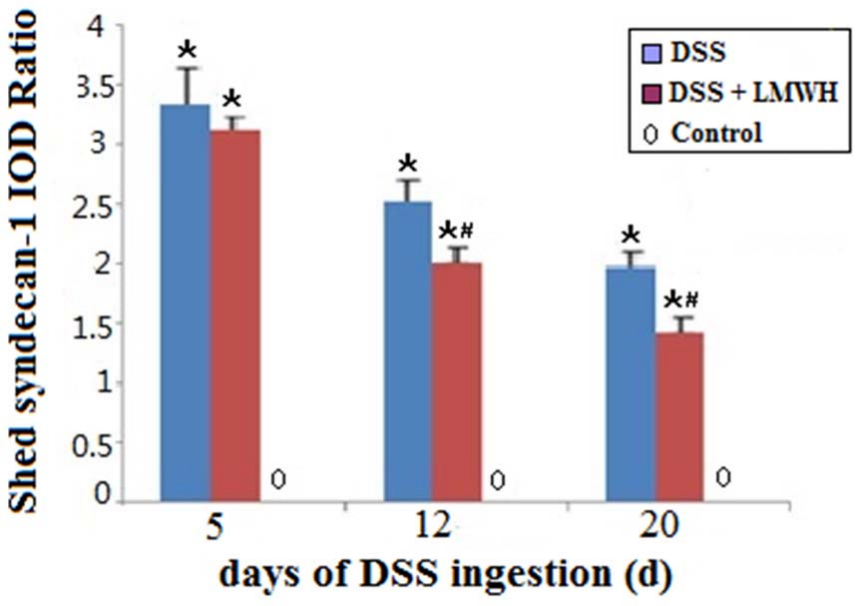
The serum syndecan-1 level. The serum syndecan-1 level in mice from different groups was examined on days 5, 12, and 20 by dot immunoassays (n = 18/group). *P*<0.05 vs. the control; #*P*<0.05 vs. the DSS group.

The dot immunoassay results further demonstrated that the level of shed syndecan-1 in the serum was significantly greater in the DSS and DSS plus LMWH groups than in the control group (*P*<0.05, Fig. 5). Moreover, in the DSS plus LMWH group, the level of shed syndecan-1 in the serum was significantly less on days 12 and 20 than that in the DSS group (*P*<0.05, Fig. 5).

We performed Spearman rank correlation analysis between syndecan-1 immunohistochemical scoring and epithelial cell damage (one of the histological scoring indicators) for their relationship in the DSS and DSS plus LMWH groups. Our result showed that their correlation was significant on days 12 and 20 after LMWH treatment compared with the DSS group (p = 0.001). Taken together, these results indicate the loss of syndecan-1 and intestinal mucosal damage in the colonic mucosa of DSS-induced colitis, while the loss of syndecan-1 and histological lesions could be restored partially by LMWH treatment.

### LMWH inhibits the expression of IL-1β and IL-10 in the intestinal mucosa of DSS-induced colitis

**Figure 6:**
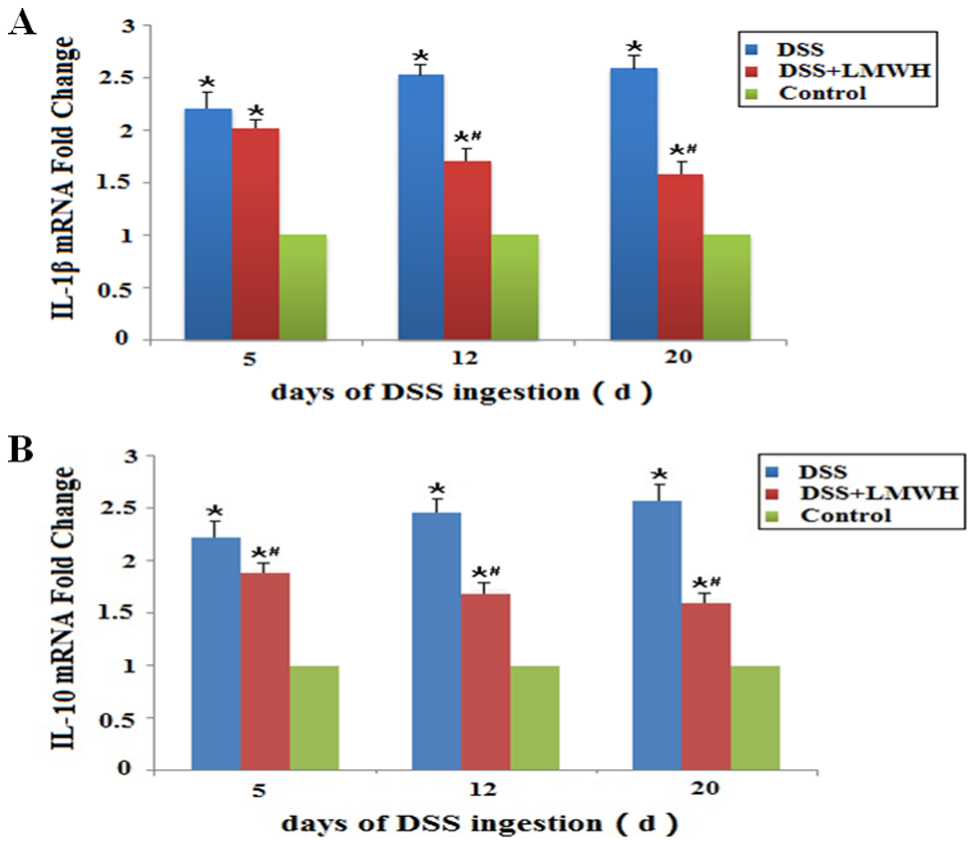
mRNA expression of IL-1β and IL-10 in the intestinal mucosa. The mRNA levels of IL-1β (A) and IL-10 (B) in the intestinal mucosa were detected by qRT-PCR on the indicated day, respectively (n = 18/group). *P<0.05 vs. the control; #*P*<0.05 vs. the DSS group.

To investigate the role of inflammatory factors in DSS-induced colitis, we determined the mRNA levels of IL-1β and IL-10 in the intestinal mucosa by qRT-PCR. In the DSS and DSS plus LMWH groups, the mRNA levels of IL-1β and IL-10 in the intestinal mucosa were significantly upregulated compared with the control group after DSS administration (*P*<0.05, Fig. 6). However, LMWH significantly downregulated both IL-1β and IL-10 mRNA levels on days 12 and 20 after LMWH treatment compared with the DSS group (*P*<0.05, Fig. 6).

We also performed Spearman rank correlation analysis between the mRNA levels of IL-1β and syndecan-1 for their relationship in the DSS and DSS plus LMWH groups. Their correlation was significant on days 12 and 20 after LMWH treatment compared with the DSS group (*P*<0.001).

## Discussion

The DSS mouse model of experimental colitis has been widely used to investigate the pathological changes of colitis and to develop effective therapeutic approaches [15], [19]. In this study, we fed C57BL/6 mice with 3% DSS solution for 5 days, followed by distilled water for 2 weeks, and established an ideal colitis model in which clinical and pathological changes were similar to those of human colitis. Acute clinical symptoms (diarrhea, guaiac-positivity, and/or grossly bloody stool) appeared within five days after DSS treatment, which were associated with crypt damage, goblet cell loss, ulcers, neutrophil infiltration of the epithelium and lamina propria, and elevated DAI values. Animals treated with DSS for 5 days, followed by 14 days of water, developed chronic colitis with the following features: loose stools, crypt and goblet cell regeneration, gland repair, and lymphocyte and monocyte infiltration of the mucosa and submucosa. These results suggested that DSS could induce acute and chronic colitis accompanied with inflammatory responses in the mice.

We found that DAI, histological score, and mRNA levels of IL-1β and IL-10 in the DSS and the DSS plus LMWH groups were greater than those in the control group. Meanwhile, the syndecan-1 mRNA and protein levels in the DSS and the DSS plus LMWH groups were less than those in the control group. Surprisingly, the level of shed syndecan-1 in the serum of the DSS and the DSS plus LMWH groups was greater than that in the control group. In the DSS plus LMWH group, LMWH could ameliorate the disease activity, relieve histopathological disturbances of DSS-induced colitis, inhibit syndecan-1 shedding, and increase the protein and mRNA levels of syndecan-1. Furthermore, LMWH downregulated the mRNA expression of inflammatory cytokines IL-1β and IL-10.

Syndecan-1 is localized physiologically on the cell-surface and in the extracellular matrix and plays critical roles in composing tight junctions and maintaining mucosal barrier function [9]. Syndecan-1 acts as a coreceptor for several tyrosine kinase receptors. For example, it reinforces the activity of the complex of basic fibroblast growth factor (bFGF) and the FGF receptor as well as promotes wound healing through stimulation of keratinocyte proliferation [7], [20]. *In vitro*, shedding of the extracellular domain of syndecan in intestinal epithelial cells disrupts FGF-dependent proliferation. In syndecan-1-deficient mice after colitis induction, delayed skin and corneal wound healing, functionally adverse repair, prolonged recruitment of inflammatory cells, and significant upregulation of TNFα were detected [6].

Furthermore, free syndecan-1 ectodomains, which contain intact HS chains, play a role in protecting the integrity of the intestinal epithelial barrier as well as syndecan-1 [6]. Since epithelial barrier function is essential for the pathogenesis of inflammatory bowel disease [21], lacking the protective factor syndecan-1 in the intestine can promote inflammatory reactions. In addition, loss of syndecan-1 expression may reduce ligand-dependent activation of growth factor receptors, thus impairing mucosal healing [22]. We found that syndecan-1^'^s mRNA and protein expression in the colonic mucosa of DSS-induced colitis mice was gradually decreased, while the levels of IL-1β and the free syndecan-1 ectodomain in the serum were increased. These results suggest that syndecan-1 shedding might be correlated with increased IL-1β levels and lead to severity of DSS colitis.

As important pro-inflammatory factors, IL-1β and TNF-a secreted by mononuclear cells in lamina propria increased in IBD tissue [23]. IL-1β can promote the activation, proliferation, differentiation of immunocytes, and the expression of immunological molecules [24]. IL-10 is an important anti-inflammatory factor secreted by mononuclear cells and macrophages in colitis, which can suppress inflammatory reaction and decrease the secretion of pro-inflammatory cytokines such as IL-1β and TNF-a. Mice deficient in IL-10 develop colitis spontaneously, and low levels of IL-10 are positively correlated with recurrences of Crohn's disease [25], [26].

The expression of syndecan-1 and the proinflammatory cytokine TNFα has been found to be inversely correlated in the colonic mucosa of patients with Crohn's disease [27]. In addition, a reduction of syndecan-1 expression has been shown to upregulate TNFα signaling in an *in vitro* model [28], [29]. Conversely, increased TNFα levels and activity induce the downregulation of syndecan-1 and promote its shedding [22], [28]. In this study, we found that the mRNA expression of IL-1β was inversely correlated with syndecan-1 in the intestinal mucosa of DSS-induced colitis. These data indicate that IL-1β, as well as TNFα, could down-regulate syndecan-1 expression [22] and may account partially for the reduced expression of syndecan-1 in DSS-induced colitis. The possible mechanism is that IL-1β can increase the synthesis of matrixmetalloproteinase-7, the sheddase of syndecan-1 [30].

It has been shown that LMWH can efficiently prevent concanavalin A-induced hepatitis in mice by inhibiting inflammation [31]. Furthermore, LMWH ameliorates the inflammatory response of experimental colitis in syndecan-1-deficient mice [32]. Previous studies have demonstrated that heparin downregulates the production of proinflammatory cytokines including IL-1β, TNFα, IL-6, and IL-8 [32], [33], [34], [35]. In this study, we found that LMWH downregulated the levels of IL-1β and IL-10 in colitis mucosa, reduced the shedding of syndecan-1, and ameliorated colitis induced by DSS. The exact anti-inflammatory mechanisms of LMWH remain unclear. We presumed that LMWH may inhibit inflammation via the following routes.

LMWH may act on upstream cytokine pathways, reduce the expression of inflammatory cytokines (such as IL-1β and IL-10) and thus alleviate the inflammation reactions in DSS-induced colitis. The reduced expression of IL-1β might lead to the upregulation of syndecan-1 in the intestinal mucosa through reducing the synthesis of matrixmetalloproteinase-7 [30].

LMWH may inhibit syndecan-1 shedding via the inhibition of heparanase. Heparanase is an endo-beta-D-glucuronidase capable of cleaving heparan sulfate (HS) side chains, yielding soluble syndecan-1. The colonic epithelium is the major heparanase producer under chronic inflammatory conditions. In inflammation processes, heparanase is also activated and further promotes syndecan-1 shedding and weakens its functions [36]. LMWH has been shown to be efficacious in ulcerative colitis and Crohn's disease patients by inhibiting the activity of heparanase [37].

LMWH is a structural and functional analogue of the syndecan-1 heparan sulfate chains and possesses ligand binding properties similar to syndecan-1 [7], [38], [39]. For example, LMWH can be a coreceptor of FGF in epithelial cell lines [40], [41]. It has been proposed that heparan sulfate might be secreted by epithelial cells to maintain an intact intestinal barrier after the shedding of dead epithelial cells [42]. Deficiency in syndecan-1 contributes to the increased severity of DSS colitis, which promotes delayed wound repair and re-epithelialization, and the function of syndecan-1 could be restored with heparin treatment [6]. Consistent with previous results [43], we found that the mRNA and protein expression of syndecan-1 in the colonic mucosa of DSS-induced colitis was greatly decreased, while the syndecan-1 level in serum was much greater. Furthermore, in the DSS plus LMWH group, LMWH greatly increased syndecan-1 expression after DSS administration and reduced the serum syndecan-1 level, compared with the DSS group (*P*<0.05). These results indicate the involvement of syndecan-1 in DSS-induced colitis and suggest that LMWH, as a substitute of the free syndecan-1 ectodomain, might prevent or relieve colitis inflammation by inhibiting syndecan-1 shedding in the colonic mucosa. Further investigation of the role of syndecan-1 shedding in the pathogenesis of colitis is needed.

In summary, our present study provides valuable insights into the anti-inflammatory role of LMWH in the treatment of colitis. Future studies will be necessary to explore the mechanism of LMWH-mediated inhibition of syndecan-1 shedding in this process. These studies will provide evidence to support the promising application of LMWH in the treatment of colitis.

## References

[1] Wolf H (1994) Low-molecular-weight heparin. Med Clin North Am 78: 733–743. 8170267 10.1016/s0025-7125(16)30155-9

[2] Pellequer Y, Meissner Y, Ubrich N, Lamprecht A (2007) Epithelial heparin delivery via microspheres mitigates experimental colitis in mice. J Pharmacol Exp Ther 321: 726–733. 17322027 10.1124/jpet.106.117226

[3] Chande N, McDonald JW, Macdonald JK (2008) Unfractionated or low-molecular weight heparin for induction of remission in ulcerative colitis. Cochrane Database Syst Rev: CD006774. 10.1002/14651858.CD006774.pub2 18425969

[4] Luo JY, Zhong Y, Cao JC, Cui HF (2011) Efficacy of oral colon-specific delivery capsule of low-molecular-weight heparin on ulcerative colitis. Biomed Pharmacother 65: 111–117. 21227626 10.1016/j.biopha.2010.12.013

[5] Chande N, McDonald JW, Macdonald JK, Wang JJ (2010) Unfractionated or low-molecular weight heparin for induction of remission in ulcerative colitis. Cochrane Database Syst Rev: CD006774. 10.1002/14651858.CD006774.pub2 18425969

[6] Floer M, Gotte M, Wild MK, Heidemann J, Gassar ES, et al (2010) Enoxaparin improves the course of dextran sodium sulfate-induced colitis in syndecan-1-deficient mice. Am J Pathol 176: 146–157. 20008145 10.2353/ajpath.2010.080639 PMC2797877

[7] Bernfield M, Gotte M, Park PW, Reizes O, Fitzgerald ML, et al (1999) Functions of cell surface heparan sulfate proteoglycans. Annu Rev Biochem 68: 729–777. 10872465 10.1146/annurev.biochem.68.1.729

[8] Gotte M (2003) Syndecans in inflammation. FASEB J 17: 575–591. 12665470 10.1096/fj.02-0739rev

[9] Alexopoulou AN, Multhaupt HA, Couchman JR (2007) Syndecans in wound healing, inflammation and vascular biology. Int J Biochem Cell Biol 39: 505–528. 17097330 10.1016/j.biocel.2006.10.014

[10] Hayashida K, Parks WC, Park PW (2009) Syndecan-1 shedding facilitates the resolution of neutrophilic inflammation by removing sequestered CXC chemokines. Blood 114: 3033–3043. 19638625 10.1182/blood-2009-02-204966 PMC2756208

[11] Fitzgerald ML, Wang Z, Park PW, Murphy G, Bernfield M (2000) Shedding of syndecan-1 and -4 ectodomains is regulated by multiple signaling pathways and mediated by a TIMP-3-sensitive metalloproteinase. J Cell Biol 148: 811–824. 10684261 10.1083/jcb.148.4.811 PMC2169376

[12] Andrian E, Grenier D, Rouabhia M (2006) Porphyromonas gingivalis gingipains mediate the shedding of syndecan-1 from the surface of gingival epithelial cells. Oral Microbiol Immunol 21: 123–128. 16476022 10.1111/j.1399-302X.2006.00248.x

[13] Pruessmeyer J, Martin C, Hess FM, Schwarz N, Schmidt S, et al (2010) A disintegrin and metalloproteinase 17 (ADAM17) mediates inflammation-induced shedding of syndecan-1 and -4 by lung epithelial cells. J Biol Chem 285: 555–564. 19875451 10.1074/jbc.M109.059394 PMC2804204

[14] Melgar S, Karlsson A, Michaelsson E (2005) Acute colitis induced by dextran sulfate sodium progresses to chronicity in C57BL/6 but not in BALB/c mice: correlation between symptoms and inflammation. Am J Physiol Gastrointest Liver Physiol 288: G1328–1338. 15637179 10.1152/ajpgi.00467.2004

[15] Cooper HS, Murthy SN, Shah RS, Sedergran DJ (1993) Clinicopathologic study of dextran sulfate sodium experimental murine colitis. Lab Invest 69: 238–249. 8350599

[16] Murano M, Maemura K, Hirata I, Toshina K, Nishikawa T, et al (2000) Therapeutic effect of intracolonically administered nuclear factor kappa B (p65) antisense oligonucleotide on mouse dextran sulphate sodium (DSS)-induced colitis. Clin Exp Immunol 120: 51–58. 10759763 10.1046/j.1365-2249.2000.01183.x PMC1905625

[17] Maitra A, Ashfaq R, Gunn CR, Rahman A, Yeo CJ, et al (2002) Cyclooxygenase 2 expression in pancreatic adenocarcinoma and pancreatic intraepithelial neoplasia: an immunohistochemical analysis with automated cellular imaging. Am J Clin Pathol 118: 194–201. 12162677 10.1309/TPG4-CK1C-9V8V-8AWC

[18] Livak KJ, Schmittgen TD (2001) Analysis of relative gene expression data using real-time quantitative PCR and the 2(-Delta Delta C(T)) Method. Methods 25: 402–408. 11846609 10.1006/meth.2001.1262

[19] Gaudio E, Taddei G, Vetuschi A, Sferra R, Frieri G, et al (1999) Dextran sulfate sodium (DSS) colitis in rats: clinical, structural, and ultrastructural aspects. Dig Dis Sci 44: 1458–1475. 10489934 10.1023/a:1026620322859

[20] Stepp MA, Gibson HE, Gala PH, Iglesia DD, Pajoohesh-Ganji A, et al (2002) Defects in keratinocyte activation during wound healing in the syndecan-1-deficient mouse. J Cell Sci 115: 4517–4531. 12414997 10.1242/jcs.00128

[21] Wehkamp J, Koslowski M, Wang G, Stange EF (2008) Barrier dysfunction due to distinct defensin deficiencies in small intestinal and colonic Crohn's disease. Mucosal Immunol 1 Suppl 1S67–74. 19079235 10.1038/mi.2008.48

[22] Day RM, Mitchell TJ, Knight SC, Forbes A (2003) Regulation of epithelial syndecan-1 expression by inflammatory cytokines. Cytokine 21: 224–233. 12824007 10.1016/s1043-4666(03)00091-7

[23] Reinecker HC, Steffen M, Witthoeft T, Pflueger I, Schreiber S, et al (1993) Enhanced secretion of tumour necrosis factor-alpha, IL-6, and IL-1 beta by isolated lamina propria mononuclear cells from patients with ulcerative colitis and Crohn's disease. Clin Exp Immunol 94: 174–181. 8403503 10.1111/j.1365-2249.1993.tb05997.x PMC1534387

[24] Youngman KR, Simon PL, West GA, Cominelli F, Rachmilewitz D, et al (1993) Localization of intestinal interleukin 1 activity and protein and gene expression to lamina propria cells. Gastroenterology 104: 749–758. 8440434 10.1016/0016-5085(93)91010-f

[25] Kuhn R, Lohler J, Rennick D, Rajewsky K, Muller W (1993) Interleukin-10-deficient mice develop chronic enterocolitis. Cell 75: 263–274. 8402911 10.1016/0092-8674(93)80068-p

[26] Meresse B, Rutgeerts P, Malchow H, Dubucquoi S, Dessaint JP, et al (2002) Low ileal interleukin 10 concentrations are predictive of endoscopic recurrence in patients with Crohn's disease. Gut 50: 25–28. 11772962 10.1136/gut.50.1.25 PMC1773067

[27] Principi M, Day R, Marangi S, Burattini O, De Francesco V, et al (2006) Differential immunohistochemical expression of syndecan-1 and tumor necrosis factor alpha in colonic mucosa of patients with Crohn's disease. Immunopharmacol Immunotoxicol 28: 185–195. 16873088 10.1080/08923970600815048

[28] Bode L, Eklund EA, Murch S, Freeze HH (2005) Heparan sulfate depletion amplifies TNF-alpha-induced protein leakage in an in vitro model of protein-losing enteropathy. Am J Physiol Gastrointest Liver Physiol 288: G1015–1023. 15604198 10.1152/ajpgi.00461.2004

[29] Bode L, Murch S, Freeze HH (2006) Heparan sulfate plays a central role in a dynamic in vitro model of protein-losing enteropathy. J Biol Chem 281: 7809–7815. 16434407 10.1074/jbc.M510722200

[30] Klein RD, Borchers AH, Sundareshan P, Bougelet C, Berkman MR, et al (1997) Interleukin-1beta secreted from monocytic cells induces the expression of matrilysin in the prostatic cell line LNCaP. J Biol Chem 272: 14188–14192. 9162049 10.1074/jbc.272.22.14188

[31] Hershkoviz R, Bruck R, Aeed H, Shirin H, Halpern Z (1999) Treatment of concanavalin A-induced hepatitis in mice with low molecular weight heparin. J Hepatol 31: 834–840. 10580580 10.1016/s0168-8278(99)80284-0

[32] McBride WT, Armstrong MA, McMurray TJ (1996) An investigation of the effects of heparin, low molecular weight heparin, protamine, and fentanyl on the balance of pro- and anti-inflammatory cytokines in in-vitro monocyte cultures. Anaesthesia 51: 634–640. 8758154 10.1111/j.1365-2044.1996.tb07844.x

[33] Chowers Y, Lider O, Schor H, Barshack I, Tal R, et al (2001) Disaccharides derived from heparin or heparan sulfate regulate IL-8 and IL-1 beta secretion by intestinal epithelial cells. Gastroenterology 120: 449–459. 11159885 10.1053/gast.2001.21202

[34] Baram D, Rashkovsky M, Hershkoviz R, Drucker I, Reshef T, et al (1997) Inhibitory effects of low molecular weight heparin on mediator release by mast cells: preferential inhibition of cytokine production and mast cell-dependent cutaneous inflammation. Clin Exp Immunol 110: 485–491. 9409655 10.1046/j.1365-2249.1997.4541471.x PMC1904823

[35] Koller M, Kutscha-Lissberg F, Brom J, Weidinger G, Muhr G (2001) Influence of low molecular weight heparin (certoparin) and unfractionated heparin on the release of cytokines from human leukocytes. Inflammation 25: 331–337. 11820460 10.1023/a:1012883916991

[36] Waterman M, Ben-Izhak O, Eliakim R, Groisman G, Vlodavsky I, et al (2007) Heparanase upregulation by colonic epithelium in inflammatory bowel disease. Mod Pathol 20: 8–14. 17041566 10.1038/modpathol.3800710

[37] Prajapati DN, Newcomer JR, Emmons J, Abu-Hajir M, Binion DG (2002) Successful treatment of an acute flare of steroid-resistant Crohn's colitis during pregnancy with unfractionated heparin. Inflamm Bowel Dis 8: 192–195. 11979140 10.1097/00054725-200205000-00006

[38] Bishop JR, Schuksz M, Esko JD (2007) Heparan sulphate proteoglycans fine-tune mammalian physiology. Nature 446: 1030–1037. 17460664 10.1038/nature05817

[39] Yip GW, Smollich M, Gotte M (2006) Therapeutic value of glycosaminoglycans in cancer. Mol Cancer Ther 5: 2139–2148. 16985046 10.1158/1535-7163.MCT-06-0082

[40] Day R, Forbes A (1999) Heparin, cell adhesion, and pathogenesis of inflammatory bowel disease. Lancet 354: 62–65. 10406379 10.1016/S0140-6736(98)09267-8

[41] Nikolova V, Koo CY, Ibrahim SA, Wang Z, Spillmann D, et al (2009) Differential roles for membrane-bound and soluble syndecan-1 (CD138) in breast cancer progression. Carcinogenesis 30: 397–407. 19126645 10.1093/carcin/bgp001

[42] Watson AJ, Chu S, Sieck L, Gerasimenko O, Bullen T, et al (2005) Epithelial barrier function in vivo is sustained despite gaps in epithelial layers. Gastroenterology 129: 902–912. 16143130 10.1053/j.gastro.2005.06.015

[43] Wang X, Chen Y, Song Y, Zhang S, Xie X (2011) Activated Syndecan-1 shedding contributes to mice colitis induced by dextran sulfate sodium. Dig Dis Sci 56: 1047–1056. 20936359 10.1007/s10620-010-1398-8

